# Senolysis potentiates endothelial progenitor cell adhesion to and integration into the brain vasculature

**DOI:** 10.1186/s13287-024-04042-2

**Published:** 2024-11-11

**Authors:** Tri Duc Lam, István Tóth, Anca Hermenean, Imola Wilhelm, Claudine Kieda, István Krizbai, Attila E. Farkas

**Affiliations:** 1 Institute of Biophysics, HUN-REN Biological Research Centre, Szeged, 6726 Hungary; 2 Doctoral School of Biology, University of Szeged, Szeged, 6726 Hungary; 3 Foundation for the Future of Biomedical Sciences in Szeged, Szeged Scientists Academy, Szeged, 6720 Hungary; 4 Department of Cell Biology and Molecular Medicine, University of Szeged, Szeged, 6726 Hungary; 5 Institute of Life Sciences, “Vasile Goldis” Western University of Arad, Arad, 310414 Romania; 6 Laboratory of Molecular Oncology and Innovative Therapies, Military Institute of Medicine-National Research Institute, Warsaw, 04-141 Poland; 7 Centre for Molecular Biophysics, UPR 4301 CNRS, Orleans, 45071 France

**Keywords:** Brain, Ageing, Embryonic endothelial progenitor cells, Senescence, Senolysis, Navitoclax, Abt-263, Dasatinib, Quercetin, Hypoxia, Vascular regeneration

## Abstract

**Background:**

One of the most severe consequences of ageing is cognitive decline, which is associated with dysfunction of the brain microvasculature. Thus, repairing the brain vasculature could result in healthier brain function.

**Methods:**

To better understand the potential beneficial effect of endothelial progenitor cells (EPCs) in vascular repair, we studied the adhesion and integration of EPCs using the early embryonic mouse aorta–gonad–mesonephros – MAgEC 10.5 endothelial cell line. The EPC interaction with brain microvasculature was monitored ex vivo and in vivo using epifluorescence, laser confocal and two-photon microscopy in healthy young and old animals. The effects of senolysis, EPC activation and ischaemia (two-vessel occlusion model) were analysed in BALB/c and FVB/Ant: TgCAG-yfp_sb #27 mice.

**Results:**

MAgEC 10.5 cells rapidly adhered to brain microvasculature and some differentiated into mature endothelial cells (ECs). MAgEC 10.5-derived endothelial cells integrated into microvessels, established tight junctions and co-formed vessel lumens with pre-existing ECs within five days. Adhesion and integration were much weaker in aged mice, but were increased by depleting senescent cells using abt-263 or dasatinib plus quercetin. Furthermore, MAgEC 10.5 cell adhesion to and integration into brain vessels were increased by ischaemia and by pre-activating EPCs with TNFα.

**Conclusions:**

Combining progenitor cell therapy with senolytic therapy and the prior activation of EPCs are promising for improving EPC adhesion to and integration into the cerebral vasculature and could help rejuvenate the ageing brain.

## Background

Endothelial cells lining the interior surface of the brain vasculature have very specific roles that differentiate these cells from the endothelium of other organs. The central nervous system endothelium forms the basis of the blood-brain barrier (BBB), which maintains almost complete separation of the brain from the rest of the body while managing bidirectional transport of nutrients and waste. Ageing and age-related pathologies profoundly affect brain vessel morphology and function [1, 2]. Thus, understanding and treating age-related deficiencies and pathologies is increasingly important. In 2020 the number of people over 65 years old was 727 million and this number is anticipated to more than double by the year 2050, meaning that one in every six people will be over the age of 65 [3].

At the tissue level, ageing causes an increase in the number of senescent cells. Senescence is a protective mechanism against tumour formation that prevents the proliferation of cells with damaged genomes but becomes pathologic when senescent cells accumulate. Senescent cells are irreversibly arrested in replication, are apoptosis resistant and secrete proinflammatory mediators that cause tissue inflammation and tissue damage. As these cells do not undergo apoptosis, the immune system is responsible for clearing them. However, with ageing the number of senescent cells increases [4]. The endothelium is no exception to this. Endothelial senescence is associated with endothelial dysfunction, arterial stiffening and remodelling, impaired angiogenesis, defective vascular repair, and with an increasing prevalence of atherosclerosis [5]. At the level of the brain microvasculature, increased capillary diameters and decreased capillary density paired with increased red blood cell velocities were observed during ageing [6, 7]. There is mounting evidence that reversing these deleterious changes could be achieved through vascular repair. The number of senescent cells in the ageing brain can be decreased by senolysis resulting in improved BBB properties and decreased neuroinflammation [8–10]. The injection of endothelial progenitor cells into the circulation can improve neurogenesis, possibly through neovascularisation and improved BBB properties [11–13]. We theorise that the vascular regenerative effects of senotherapy could be synergistically amplified by combining it with EPC therapy.

Senolysis, the clearance of senescent cells, was made possible by small-molecule drugs introduced in the mid-2010s that target the molecular pathways responsible for the apoptosis resistance of senescent cells [14]. Among these senolytics the antileukaemia drug, tyrosine kinase inhibitor dasatinib and the plant flavonoid quercetin were the first [15], shortly followed by the Bcl inhibitor navitoclax (abt-263) [16], which has been demonstrated to extend the lifespan of mice [17]. There are several promising applications of senolytics for improving the function of the nervous system. For example senotherapy can improve vasomotor function in atherosclerotic mice [18], ameliorate Alzheimer’s disease symptoms [19–21], is neuroprotective following brain hypoxia [22], or can rescue paclitaxel-induced microvascular senescence and attenuate chemotherapy-induced cognitive impairment [23]. Multiple clinical trials are ongoing [24], of which two phase I clinical trials ended in 2023 with one focusing on brain function [25, 26].

Research on progenitor cell therapy targeting the vasculature received a boost with the isolation of putative progenitor endothelial cells by Asahara and colleagues [27]. The wide interest in researching progenitor cells that can be mobilised to repair pathological vessels and reverse characteristic hypoxia-dependent disease consequences led to the coining of the term: “endothelial progenitor cells” or EPCs. The increased interest has also led to a variety of cell types called EPCs [28]. Authors searching to define EPCs that are able to ensure vessel repair in pathologies, have led to disputes regarding their definitions without considering the plasticity of ECs as a function of the microenvironment [29], and consequently the biological state [30]. For this reason, attempts to define the type and phenotype of EPCs recruited from the bone marrow that are able to respond to damage caused by diseases associated with angiogenic stress are still controversial. Injected exogenous EPCs can increase angiogenesis and neurogenesis and improve neural function following ischaemic injury and can even improve blood-brain barrier properties [12]. However, only a few studies have provided information about EPCs homed into the brain, resulting in a dearth of information regarding what happens with these cells over time.

Determining the specific functional properties of the human brain microvasculature is challenging, thus most of our knowledge comes from in vitro experiments or mouse models. Given that there are similarities in the gene expression and coarse organisation of the human and mouse brain [31], and that the same cell types constitute the brain microvasculature in both species [32], we assume that studies of mouse brain microvessels and EPCs can be relevant for medical research. This is further strengthened by studies in which human cells were successfully used in mouse models [33, 34].

One of the main goals of this research was to study the dynamics of EPC-brain vasculature interactions under physiological conditions, during ageing and in response to ischaemic injury. The other main goal was to study whether senolytic pretreatment using abt-263 or the combination of dasatinib and quercetin has an effect on such interactions. We used an immortalised cell line generated from early embryonic mouse aorta–gonad–mesonephros endothelial cells (MAgEC 10.5). It was selected as an early embryonic EPC line among lines obtained from murine embryos at 10.5 days post conception (dpc) and 11.5 dpc corresponding to endothelial lineage commitment during murine embryonic development [35, 36]. This cell line was developed to deliver therapeutics by homing in on the developing vasculature of tumours [35], and was further shown to be able to home into brain vasculature and deliver therapeutic molecules [37]. As a cell line, it is amenable to genetic modification making it a great tool for EPC research.

## Materials and methods

### Chemicals

Unless otherwise noted, chemicals were purchased from Sigma-Aldrich (Burlington, Massachusetts, United States).

### Cell culture

MAgEC 10.5 and tdTomato-expressing MAgEC 10.5 cells (C. Kieda Patents Nr 99-16169, WO-9631178B2) were described previously [35, 37]. Briefly, the MAgEC 10.5 cell line displaying tdTomato expression was made using the third-generation lentiviral system consisting of pMDLg/pRRE, pRSV-Rev, pMD2.G (a gift from Didier Trono (Addgene plasmid # 12251, 12253, 12259, Watertown, MA, USA)) and the expression plasmid pLV [3Exp]-EF1A>{tdTomato}:IRES: Puro (VectorBuilder, Chicago, IL, USA). Lentiviral vectors were produced using Lenti-X™ 293T cell line (Clontech, Takara, Kusatsu, Japan), according to the protocol described by Rossowska et al. [38]. Stable MAgEC 10.5/tdTomato cell line, renamed MAgEC 10.5-tomato was obtained after selection with puromycin (10 µg/mL, from Sigma-Aldrich, St. Louis, MO, USA). The transduction efficacy was analysed for the fluorescence emission of the tdTomato protein in cells by flow cytometry (FACS Aria, Becton Dickinson, Franklin Lakes, New Jersey, USA).

MAgEC 10.5 cells were cultured in OPTIMEM I supplemented with 2% FBS (Thermo Fisher Scientific, Gibco, Waltham, Massachusetts USA) in a 5% CO_2_ incubator at 37 °C. Media was changed every 2–3 days depending on cell numbers. Cultures were split at or before reaching confluence. For intracarotid injections, cells were seeded at a low density (500 000 cells per 3.5 cm diameter culture dish) the day before to ensure that cells were not clumped after trypsinisation. For passage and cell collection, 0.05% trypsin was used for 3 min. After trypsin was stopped, a small volume was used for counting cells in a haemocytometer. The rest was centrifuged for 5 min at 4 °C at 700 RCF and suspended in Ringer-HEPES (150 mM NaCl, 5.2 mM KCl, 2.2 mM CaCl_2_, 0.2 mM MgCl_2_, 6 mM NaHCO_3_, 5 mM HEPES and 2.8 mM D-glucose, pH 7,4) so that 200 µL contained 400 000 cells.

### TNFα pretreatment of MAgEC 10.5-tomato cells

MAgEC 10.5-tomato cells were seeded at 500 000 cells per 3.5 cm diameter culture dish. The following day the cells were treated with 1 or 5 ng/mL rmTNFα (Peprotech, Margravine Road, London, UK) for 1 h in serum-free OPTIMEM I prior to injection into the carotid artery of mice. To minimise negative effects of TNFα, we selected these low doses and short-treatment of the EPCs over systemic treatment of the mice.

### Animals

Young, 8–12-week-old and old, 20–30-month-old female mice were used for ageing experiments and 8–12-week-old mixed sex for other experiments. Mouse strains used were: BALB/c and FVB/Ant: TgCAG-yfp_sb #27. Animal housing conditions complied with the laws of Hungary (article 40/2013. (II. 14.)), which conforms to European Union regulations: 22 ± 2°C, 55 ± 10% humidity, 25x air change per hour, 12–12 hours light-dark cycles with ad libitum access to regular chow and water. Cardboard tunnels were used for environmental enrichment. Procedures conformed to widely accepted standards, best practices and Hungarian laws of animal protection. The primary endpoint was MAgEC 10.5 integration into brain vasculature. Group sizes were estimated using G*power when designing the experiments ([39] version 3.1.9.7) Briefly: to compare treatment groups, test family was set to “F tests”, the statistical test used was “ANOVA: Fixed effects, omnibus, one-way”, the type of analysis was “A priori: compute required sample size - given α, power and effect size. Effect size was determined from variances with “variance explained by special effect” set to 0.1 and “variance within group” set to 0.25. When comparing treatment groups over time, the statistical test used was “ANOVA: Repeated measures, within, between interactions”. For old mice, a 25% death rate was estimated. Individual mice were assigned to a list randomly, numbered for identification, and then placed into treatment groups. Experiments were not blinded, as individual experiments and their analysis was done by the same researchers. During the experiments, no confounders were identified. Animals were monitored daily for any indications for terminating the experiment such as infections, lethargy, greater than 25% body weight etc. In senolysis experiments, the number of young mice for 24 hours treatment were 6, 9, 7 while for 48 hours treatment were 7, 6 and 5 for control, abt-263 and dasatinib with quercetin groups respectively. Old groups had 5, 6, 5 mice for 24 hours treatments and 5, 6, 6 mice for 48 hours. EPC integration was checked in 2, 4 and 3 mice in the same groups as above after 5 days. For two-vessel occlusion experiments, 5 groups of BALB/c animals were used with 3 animals in each of the 5 groups. TTC staining required 5 groups (MAgEC 10.5 injected and uninjected groups at two time points and a sham surgery group) and two groups were used to assess EPC integration. For in vivo BBB permeability, 15 MAgEC 10.5 cells were observed in 7 young mice and 5 cells in 3 old mice. For in vivo determination of EPC preactivation by TNFα 6 groups were used with 3 mice each (two time points with three treatment groups). A total of 105 BALB/c and 28 FVB/Ant: TgCAG-yfp_sb #27 mice were used. Euthanasia was carried out with the isoflurane overdose method by setting the evaporator to over 5% until breathing stopped.

### Senolysis

Young and old BALB/c mice were treated with senolytics dissolved in the vehicle mixture: 60% Phosal 50 PG (Lipoid AG), 30% polyethylene glycol 400, 10% ethanol (Molar Chemicals Kft, Budapest, Hungary). One treatment regime was 100 mg/kg abt-263 (MedChemExpress, Monmouth Junction, New Jersey USA) once per day for 5 days via oral gavage, the second treatment regime was 5 mg/kg dasatinib (Sellekchem, Houston, Texas USA) combined with 50 mg/kg quercetin in a single gavage. Controls were treated with vehicle. Seven days after treatments started, mice were injected with MAgEC 10.5-tomato cells through the internal carotid artery.

### Cranial window

Cranial windows were implanted similar to previously described [40]. Anaesthesia in mice was induced with 4% isoflurane and maintained with 1–2% isoflurane using an isoflurane evaporator (RWD, Guangdong, China). Mice were placed on a heating pad and fixed to a Standard Stereotaxic Instrument (RWD, Guangdong, China). The skin was disinfected with 70% ethanol followed by iodine solution. The skin was removed, the periosteum was treated with 1% Lidocaine and then removed as well. Using an OM-6 operating microscope (Takagi, Tokyo, Japan), craniotomy (d = 3.5 mm) was performed over the right parietal cortex with a micro drill (H.MH-170, Foredom, Blackstone Industries, Bethel, Connecticut, USA) fitted with a 0.5 mm burr, followed by the removal of the dura. The centres for the craniotomies were approximately 1.7 mm posterior and 2.2 mm lateral from bregma. Throughout the operation, the area was irrigated with sterile Ringer-HEPES. The cranial window was covered with a round coverslip (d = 5 mm) and cemented in place using acrylic glue. An aluminium head plate was cemented to the skull. After the procedure, mice injected with Rimadyl (carprofen, 5 mg/kg) i.p. and were left to convalesce to reduce inflammation from the surgery.

### Intracarotid injection

Intracarotid injections were performed as previously described [40]. Briefly, under isoflurane anaesthesia, mice were fixed to the stereotaxic frame. The fur on the neck was shaved and the skin was disinfected with 70% ethanol and iodine solution. After making a less than 1 cm cut on the skin along the neck, tissues were bluntly dissected under an OM-6 operating microscope (Takagi, Tokyo Japan), to reveal the right common carotid artery. The artery was separated from surrounding tissues with a pair of surgical forceps. Temporary ligations were made at the proximal part of the common carotid artery and at the external carotid artery. A moist cotton ball was used to lift the common carotid then 400 000 MAgEC 10.5 cells were injected suspended in 200 µL Ringer-HEPES. The ligations were opened and the wound was closed. Throughout the operation, the area was irrigated with sterile Ringer-HEPES.

### Femoral artery cannulation and fluorescent tracer injection

The inner thigh of the mouse was shaved and disinfected with 70% ethanol and iodine. The skin was cut parallel to the femur and connective tissue was retracted. Under an OM-6 operating microscope (Takagi, Tokyo Japan), the femoral artery, vein and nerve were separated bluntly. The artery was lifted from the tissue by placing three pieces of surgical thread and three ligatures were placed. The distal ligature was tightened permanently and the proximal was tightened temporarily. A small cut was made on the artery with microscissors through which the cannula was inserted and fixed in position with the middle ligation and with the remaining thread of the distal one. The proximal ligature was opened to gain access to the circulation and the skin was closed. Throughout the operation, the area was irrigated with sterile Ringer-HEPES.

### Two-vessel occlusion (2VO)

This procedure is similar to intracarotid injection described above. Using an OM-6 operating microscope (Takagi, Japan, Tokyo Japan), the common carotids were revealed on both sides. Both arteries were closed using microclamps for 30 min, then the clamps were removed and the wound was closed. When 2VO was combined with MAgEC 10.5 injection, the right side was prepared as described above for intracarotid injection while the left side was prepared as described here. Cells were injected at the end of the ischaemic period.

### Quantification of MAgEC 10.5 adhesion and immunofluorescence

Animals were transcardially perfused with phosphate-buffered saline (PBS, pH 7.4) and fixed with 4% formaldehyde in PBS. Brains were removed and postfixed in formaldehyde at 4 °C overnight than placed in PBS and kept at 4 °C. Coronal sections were made using a vibratome (VT1000 S, Leica Biosystems, Wetzlar, Germany). PBS with 0.05% sodium azide was used to preserve 30 μm brain sections. Brain sections were temporarily mounted with PBS to count MAgEC 10.5 cells, then selected sections used for immunofluorescent labelling. Sections were incubated in PBS for 20 min at 85 °C to perform antigen retrieval. 0.5% Triton X-100 in PBS was used for permeabilisation for 1 h at room temperature. Sections were then blocked with 3% bovine serum albumin (BSA) in PBS. The primary antibodies against claudin-5 (35-2500, Life Technologies Magyarország Kft., Thermo Fisher Scientific, Invitrogen, Waltham, Massachusetts USA) were incubated overnight at 4 °C in blocking solution on a rotary shaker. Sections were washed 3 times for 5 min in PBS before secondary antibody incubation for 60 min at room temperature in the dark followed by 3 times 5 min wash in PBS. Nuclei were stained with Hoechst 33342 for 5 min, washed with PBS, and mounted with FluoroMount-G medium (Southern Biotech, USA). A fluorescence microscope (Axiovert Z1, Zeiss, Budapest, Hungary), equipped for super-resolution capable laser scanning confocal microscopy (Stedycon, Abberior Instruments, Göttingen, Germany), was used to record immunofluorescence.

### Two-photon microscopy

Intravital, two-photon imaging was performed as previously described [41]. Briefly, under isoflurane anaesthesia, mice that underwent cranial window implantation were placed on a heating pad and were fixed in place using the head plates. Intravital microscopy was conducted with a FEMTO 3D Dual microscope (Femtonics, Budapest, Hungary) equipped with a 20x large working distance, water immersion objective (XLUMPLFLN-20XW, Olympus), a Mai Tai HP Ti-sapphire laser (RK TECH Ltd.) and GaAsP photomultipliers using the MES software (v6, Femtonics, Budapest, Hungary). The individual patterns of pial vessels were exploited for repeated recording of the same tissue volumes with 3 μm z-step. Image stacks were processed in FIJI (software versions: ImageJ154f and Java 1.8.0_66 64 bit) [42].

### In vivo BBB permeability measurement

Young and old FVB/Ant: TgCAG-yfp_sb #27 mice underwent cranial window implantation for two-photon microscopy. The mice were injected with MAgEC 10.5-tomato. Mice in which red fluorescent MAgECs were present within the cranial window two days after injection were then cannulated in the femoral artery, placed back into the two-photon microscope and injected with Na-fluorescein and Evans blue as low- and high-molecular-weight tracers through the cannula. Fluorescence intensity of the tracers was measured in two-photon images in regions of interest (ROI) near blood vessels containing MAgEC 10.5-tomato cells and control vessels that did not. The average distance between capillaries in the brain is approximately 40 μm, thus one ROI was chosen immediately next to the vessel, while the second one was placed 20 μm farther. The second ROI was chosen in a way that no other vessel was closer to it based on previous three-dimensional imaging.

### Quantification of MAgEC 10.5 integration

Formaldehyde-fixed 30 μm brain sections from control and 2VO mice were photographed using a fluorescence microscope (Axiovert Z1, Zeiss, Budapest, Hungary) with a digital camera and the µManager software [43, 44]. Cells were grouped based on morphology. Solid, bright cells visibly filling the vessel lumen were considered adherent. Cells showing clear endothelial morphology with thin cells surrounding the lumen with a thicker central region containing the nucleus were counted as integrated. This determination is very straightforward in vessels parallel or close to parallel with the plane of sectioning; however, the morphology of some vessels running near or at right angle with the brain section could not be determined.

### 2,3,5-triphenyltetrazolium chloride (TTC) staining

The brains were quickly isolated and placed in cold PBS (0–4 °C). The brain was sectioned into 450-µm-thick coronal slices using a vibratome (VT1000 S, Leica Biosystems, Wetzlar, Germany). Sectioning was finished within 10 min of decapitation. Brain slices were immersed in TTC solutions (10 ml, 2% TTC in PBS) for 10 min at 37 °C. Staining was followed by fixation in 4% formaldehyde solution. Metabolically active regions were stained red, whereas the cerebral infarction remained white. Sections were photographed using our Takagi OM-6 operating microscope and infarct volume was measured using FIJI.

### Statistical analysis

Data for each group were tested for normality (shapiro.test()) and for outliers (identify_outliers()), then ANOVA with post hoc Tukey test was done in R with R Commander (versions 4.3.1 and 2.8-0). T-tests were calculated in Excel (Microsoft Office 2016). Data were presented as standard box plots or bar graphs of means with standard deviations as noted in figure legends. Statistically significant effects were labelled with the level of significance. No animals or measurements were excluded from the study.

## Results

### MAgEC 10.5 EPC adhesion to the brain vasculature

**Fig. 1:**
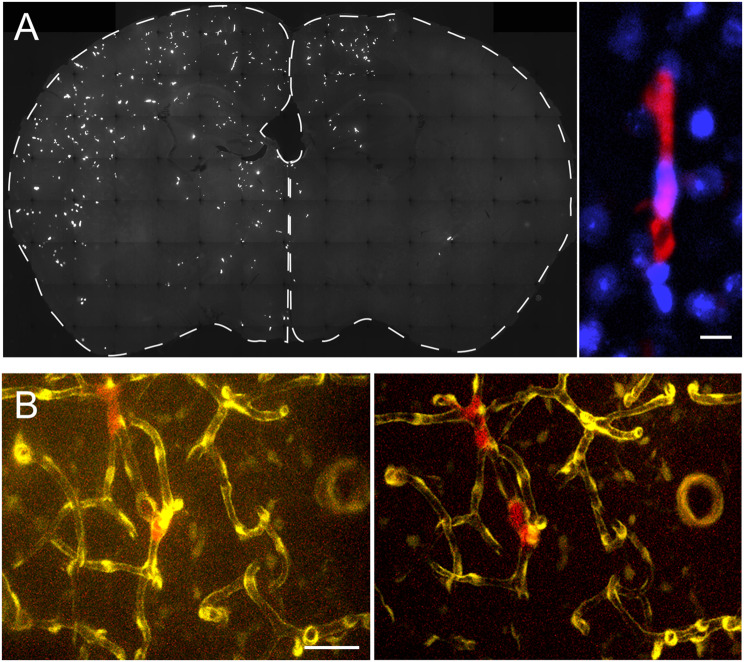
MAgEC 10.5 EPC adhesion in brain vessels **A** MAgEC 10.5-tomato cells injected in BALB/c mice were observed ex vivo in the ipsilateral hemisphere 1 day after injection. Right panel: example of an adherent red fluorescent progenitor cell. Nuclei labelled blue with Hoechst 33342. **B** MAgEC 10.5-tomato were followed in vivo, in the somatosensory cortex of FVB/Ant: TgCAG-yfp_sb #27 mice using two-photon microscopy, having vascular endothelial cells labelled in yellow. Representative progenitor cells (red) staying at the same location 24 and 28 h after injection. Scale bars are 10 μm

MAgEC 10.5-tomato cells, expressing the red fluorescent tdTomato protein and injected through the internal carotid artery (ICA) of young BALB/c mice, rapidly adhered to the brain vasculature. Even days later, most of the observed cells were in the ipsilateral hemisphere (Fig. 1A). In brain sections, the injected progenitor cells were inside capillary vessels and appeared to fill the lumen. However, the microvessels were likely not completely blocked, as we did not observe blood cells stuck next to the EPCs in the vessel lumens after transcardial perfusion or during in vivo microscopy.

By monitoring MAgEC 10.5-tomato cells through cranial windows in FVB/Ant: TgCAG-yfp_sb #27 mice, which express Venus yellow fluorescent protein in endothelial cells, we observed that at this point, some of the red fluorescent MAgEC 10.5-tomato cells remained at the same location for 24 h (Fig. 1B). Some cells were adherent to the same location for 5 days or longer (data not shown), while others detached and moved to different sites (data not shown).

### MAgEC 10.5 EPC integration into the capillary bed

**Fig. 2:**
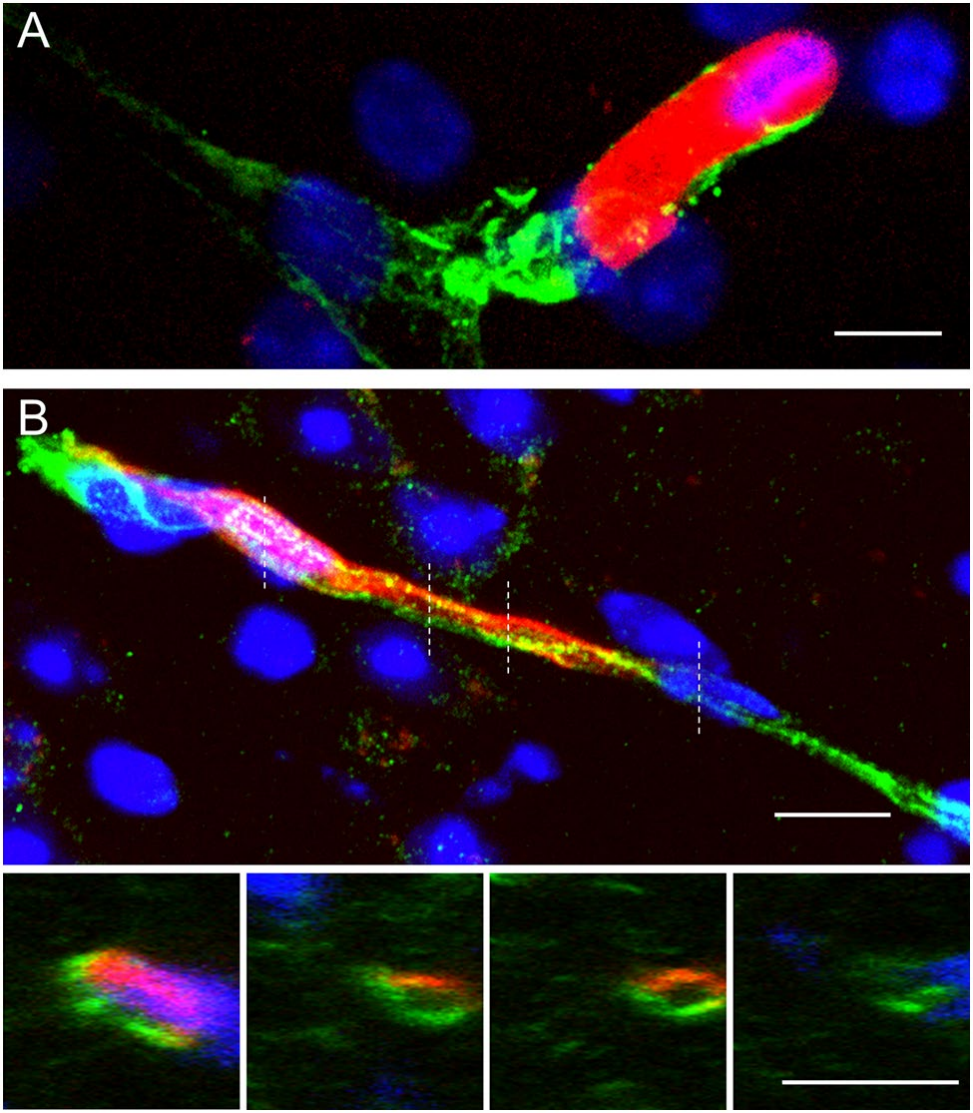
MAgEC 10.5 EPC integration into the capillary bed. MAgEC 10.5-tomato cells injected into BALB/c mice. Immunofluorescent labelling of claudin-5 in green shows: **A** MAgEC 10.5-tomato cell in the lumen of a brain microvessel and **B** MAgEC 10.5-tomato cell differentiated into an endothelial cell and integrated into the wall of a microvessel observed in the thalamus. Orthogonal images taken at the dashed lines reveal the vessel lumen formed by the MAgEC 10.5-tomato-derived endothelial cell and a pre-existing endothelial cell together. B Scale bars are 10 μm

By monitoring MAgEC 10.5-tomato cells in young BALB/c brain tissue after injection, we found that within five days some cells took up endothelial-like morphology. Immunofluorescence labelling and laser confocal microscopy revealed that MAgECs integrated into the capillary bed, presenting continuous tight junctions (TJs) visualised by claudin-5 staining and forming the vessel lumen together with pre-existing endothelial cells (Fig. 2). The existence of TJs between the MAgEC 10.5-tomato-derived and preexisting cells. The presence of a vessel lumen strongly suggest that these are functional blood vessels.

**Fig. 3:**
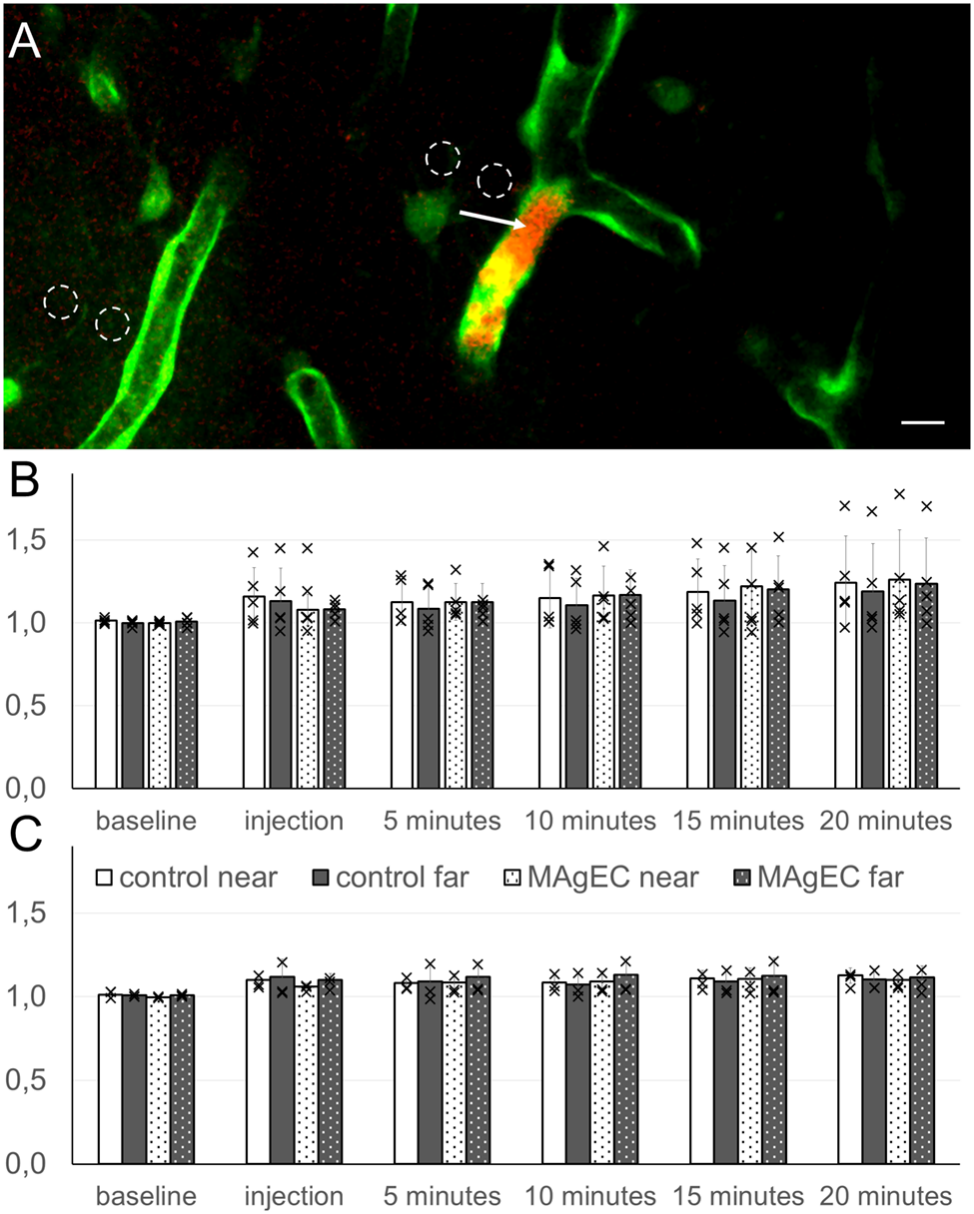
In vivo BBB permeability measurements at MAgEC 10.5 EPC adhesion sites. Two days after injecting MAgEC 10.5-tomato in FVB/Ant: TgCAG-yfp_sb #27 mice, vascular permeability was assayed in vivo near MAgEC 10.5 cells in the somatosensory cortex. **A** Example maximum intensity projection for the location of ROIs for fluorescence measurement near a MAgEC cell marked with the arrow and at a control vessel. The intensity measurements were done on single optical sections. Scale bar is 10 μm. **B** and **C** Bar graphs show relative fluorescence intensity over time next to or farther away from vessels, which either contained a MAgEC 10.5-tomato cell or not in the brain of **B** young or **C** old mice. Means +/- standard deviations are presented, *n*=5 for young and *n*=3 for old

### In vivo BBB function at EPC adhesion sites

To determine whether the arrest of EPCs induced any changes in vascular functionality, functional assessment of the BBB and TJs was performed by monitoring in vivo permeability using intravital two-photon microscopy. ANOVA analysis revealed that the BBB permeability for Na-fluorescein (Fig. 3) and Evans blue-albumin (not shown) was not significantly changed in either young or old mice two days after the injection of cells. We also did not observe a gradient between the ROI closer to or farther from the vessel in question.

### Effect of MAgEC 10.5 EPC activation, by TNFα pretreatment, on adhesion and integration in vivo

**Fig. 4:**
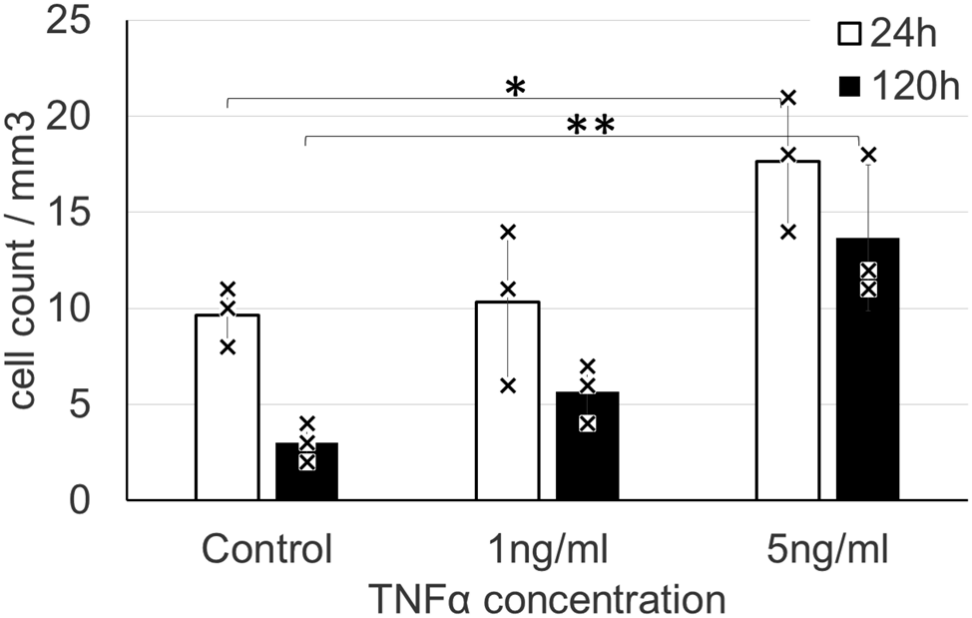
Effect of TNFα on MAgEC 10.5 EPC adhesion in vivo in young BALB/c mice. Based on intravital two-photon imaging, MAgEC 10.5-tomato cells pretreated with TNFα adhered to the vasculature in greater numbers 24 h after injection. Revisiting the same cells after 120 h revealed that more cells remained at the same location if pretreated with TNFα. Means +/- standard deviation are presented, *n*=3 for each group, ANOVA: * *p*≤0.05, ** *p*≤0.01

Theoretically, the positive effect of EPCs on tissue revascularisation can be enhanced by increasing the number of progenitors that adhere to the brain vasculature. As TNFα activates EPC adhesion in vitro [45], we pretreated MAgEC 10.5-tomato cells in culture for one hour with 1 or 5 ng/mL TNFα before injecting them into FVB/Ant: TgCAG-yfp_sb #27 mice. Using intravital two-photon microscopy, we saw an increased number of adherent cells 24 h after injection when cells were pretreated with TNFα. After 120 h, a larger number of individual cells remained at the same location in the vasculature in response to TNFα pretreatment of EPCs compared with untreated controls (Fig. 4).

### Effect of senolysis on the adhesion and integration of MAgEC 10.5 EPCs in vivo

**Fig. 5:**
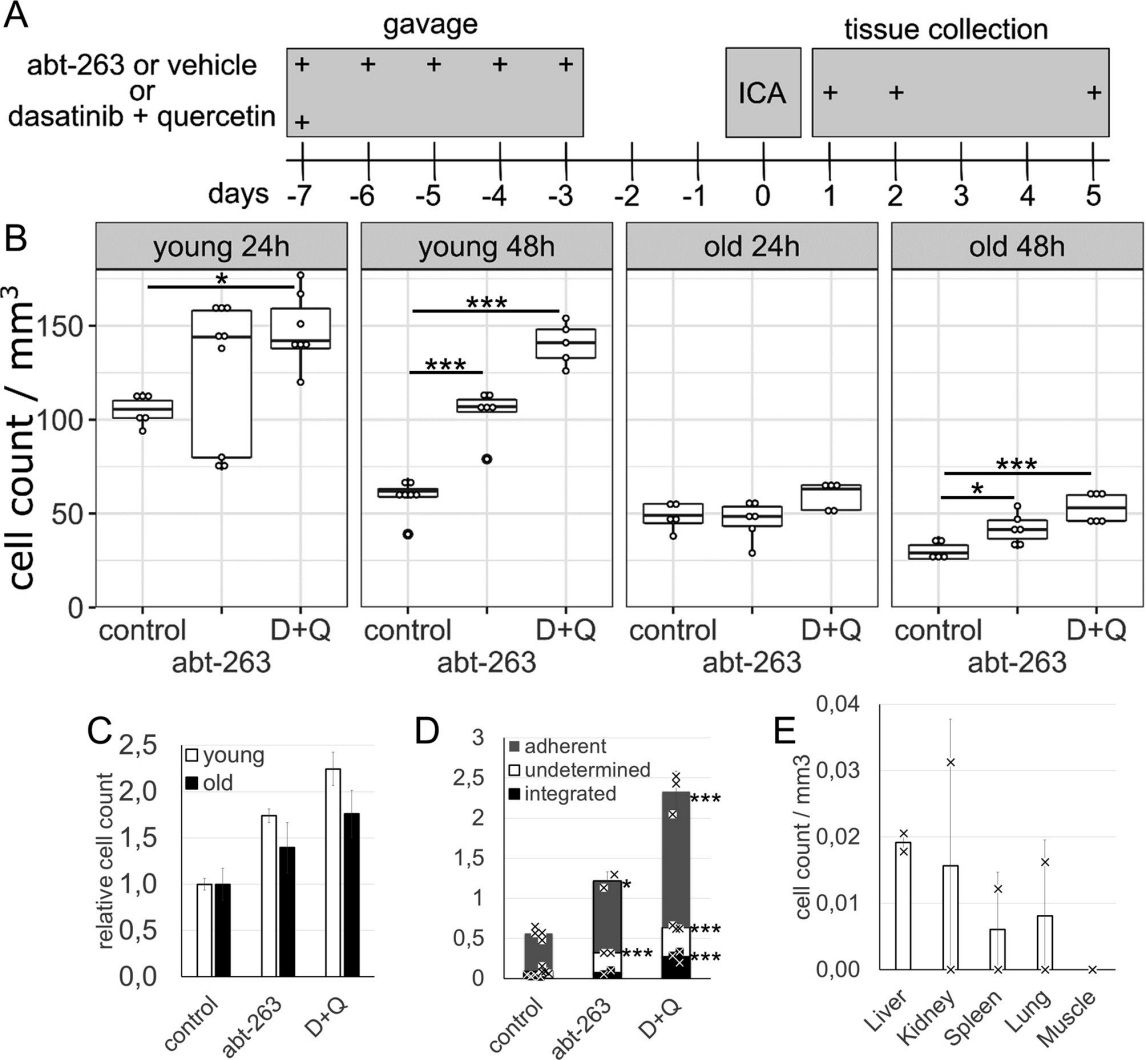
Effect of senolysis on MAgEC 10.5 EPC adhesion in young and old BALB/c mice. **A** Treatment schedules for senolytics and progenitor injections. Young and old BALB/c mice were pretreated with either abt-263 or dasatinib + quercetin (D+Q). One week later MAgEC 10.5-tomato cells were injected through the internal carotid artery. Brains were sectioned 24, 48 h or 5 days after injection. **B** Adherent progenitor cells were counted on vibratome sections. In young mice at 24 and 48 h, both types of senolytic pretreatments caused a significant increase in the number of adherent cells. In old mice, the results were similar but less pronounced: 24 h after injection there was no significant change; however, 48 h after injection both abt-263 and dasatinib + quercetin pretreatment significantly increased progenitor cell adhesion to brain capillaries. **C** Comparison of the relative effect of senolysis on 48-hour progenitor cell adhesion between young and old mice by normalizing to the adherent cell numbers of the respective controls (comparing 2^nd^ and 4^th^ panels of **B**). **D** In old mice, 5 days after injection, MAgEC 10.5-tomato cells integrated into the vasculature. Both adhesion and integration were increased by senolytic treatment. Control *n*=4, abt-263 *n*=2, D+Q *n*=3. **E** In young mice, 48 h after intracarotid injection, there were negligible numbers of adherent MAgECs in the liver, kidney, spleen, lung and muscle tissue (*n*=2). Significance values are based on ANOVA with Tukey’s post hoc test. * *p*≤0.05, *** *p*≤0.01 Bar graphs are means +- standard deviations

Using senolytic treatments in young and old BALB/c mice, we aimed to increase the number of EPC adhesion events by creating a niche for adhesion in place of the eliminated senescent endothelial cells. Mice underwent senolysis, followed by MAgEC 10.5-tomato injection, and 24–48 h later, the number of adherent cells was counted (Fig. 5A). We found that both senolytic treatments increased MAgEC 10.5 adhesion to the brain vasculature at both time points in young mice and after 48 h in old mice (Fig. 5B). Dasatinib + quercetin treatment caused a noticeably larger increase compared to abt-263. In old animals, there was a marked decrease in the number of adherent cells compared with young animals. Twenty-four hours after injection, young animals had over 100 MAgEC 10.5-tomato cells per mm^3^ brain tissue while old animals had half as much. Forty-eight hours after injection, the number of adherent cells in young animals was 63+/-4, while in old animals it was 30+/-5. Senolytic treatments were unable to completely negate the lower adhesion in old mice, even though the relative increase (compared with their respective controls) caused by the senolytic treatments was similar. At 48 h the young animals had 109+/-5 or 140+/-11 adherent cells after abt-263 or dasatinib + quercetin (D + Q) pretreatment whereas the old animals had 42+/-8 and 53+/-8, respectively. The relative effect of senolysis on EPC adhesion was only slightly less for the old mice compared with the young mice: 174%, 224% effect for abt-263 and D + Q in young animals while 140%, 176% in old animals (Fig. 5C). Given that the integration of EPCs into the vasculature took five days, we tested the effect of senolytic treatment on MAgEC 10.5 integration in old BALB/c mice at this timepoint. Even though the groups for this experiment were smaller, making these results statistically less powerful, we found a significant increase in EPC integration in the dasatinib + quercetin pretreated animals (Fig. 5D). Lastly, we checked whether the MAgEC 10.5-tomato cells reached other organs beside the brain after intracarotid injection, and found very low cells counts after two days (Fig. 5E) and none after five days (not shown).

### Effect of hypoxia on MAgEC 10.5 EPC integration in vivo

**Fig. 6:**
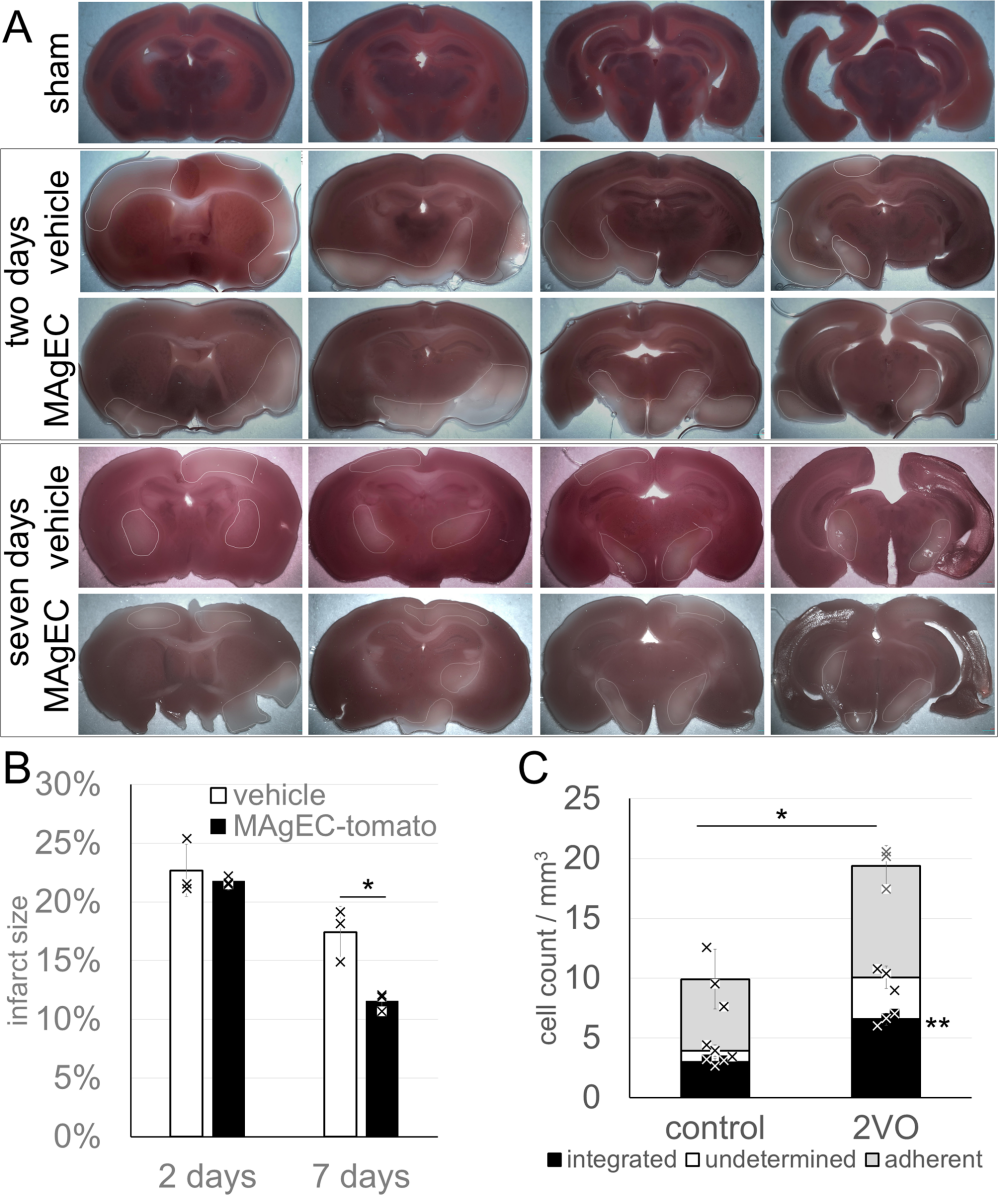
Interaction of MAgEC 10.5 cells with the brain vasculature in young BALB/c mice under experimental hypoxia (2VO). **A** Representative images of TTC staining for infarct volume. The stained area (red) is healthy whereas the unstained area (white) is the infarct area. **B** Summarised data showing the infarct volume (percentage of brain section) in treatment groups following 2VO ischemic stroke. For each group *n*=3, t-test: * *p* = 0.013. **C** The number of both adherent and integrated MAgEC 10.5-tomato increased in 2VO animals compared with controls 5 days after injection. *n*=3, 16 sections each, t-test: ** *p*=0.00782 Bar graphs are means +- standard deviations

As tissue damage induced by ischaemic injury can be alleviated by EPC treatment, we used an experimental hypoxia model to study the role of EPC integration in this process. To model hypoxia, BALB/c mice underwent two-vessel occlusion (2VO, which causes mild ischemic stroke in the anterior circulation) before being injected with MAgEC 10.5-tomato. The extent of injury was measured by comparing the area showing tissue damage to the whole area of brain sections on photographs after TTC staining (Fig. 6A). Injection of MAgEC 10.5-tomato resulted in significantly greater recovery of the ischaemic tissue. Two days after ischaemic insult, the injured brain tissue volume was 22 and 21% for sham and MAgEC 10.5-tomato injected animals, whereas after 7 days, it decreased to 17 and 11% respectively (Fig. 6B). To determine the effect of hypoxia on EPC integration, we counted MAgEC 10.5-tomato cells in tissue sections five days after 2VO and EPC injection. We observed a significant, approximately twofold increase in the total and integrated number of MAgEC 10.5-tomato cells in response to 2VO compared with sham-operated controls (Fig. 6C).

## Discussion

For healthy and long life, the health of the brain is of great importance. Ageing and ageing-associated neurovascular and neurodegenerative diseases are accompanied by dysfunction of the capillary vessel endothelium resulting in impaired function of the neurovascular unit. We explored two strategies for increasing the interactions between endothelial progenitor cells and the vasculature: increasing the available pool of endothelial progenitors by injecting EPCs into the circulation of mice and decreasing the number of senescent cells through senolysis. As EPCs, we used the early embryonic MAgEC 10.5 cell line [35] expressing the red fluorescent tdTomato to enable observation over multiple days [37].

Injected MAgEC 10.5 were almost exclusively detected in the hemisphere ipsilateral to the injection, suggesting that EPCs adhere to the vasculature shortly after injection and do not pass through the circulation multiple times. As hypoxia increases the adhesion of EPCs to the mature endothelium, this unilateral distribution could be at least partially explained by a short and slight hypoxia caused by the injection in the ipsilateral internal carotid artery, or the injected cells themselves can cause hypoperfusion in their vicinity, as shown previously [46]. In line with our results, but in Sprague Dawley rats that underwent middle cerebral artery occlusion (MCAO), Ohta et al. reported that GFP-expressing, bone marrow-derived EPCs injected through the internal carotid could be observed throughout the ipsilateral hemisphere and at the surface of arteries after 24 h [47]. The early adhesion of EPCs to brain vessels after intracarotid injection is further supported by the very low number of MAgEC 10.5 cells in peripheral tissues.

To assess the dynamics of EPC–brain vasculature interactions we followed MAgEC 10.5-tomato cells in the brains of FVB/Ant: TgCAG-yfp_sb #27 mice by intravital two-photon microscopy. In a cranial window, we could image approximately 4–9 volumes of 700*700*400 µm brain tissue. EPCs rarely remained at the same location for multiple days. In a five-day period, only approximately 3 cells out of 10 could be found again at the same location. TNFα levels are increased both during ageing and in stroke [48, 49] and TNFα treatment increased both bond number and affinity between early outgrowth EPCs and rat cardiac microvascular endothelial cells [45]. Thus, we tested the effect of TNFα pretreatment in our in vivo system and found that the number of EPCs adherent to the cortical microvasculature nearly doubled and most of the pretreated cells remained adherent to the same location for five days. Chronic exposure to TNFα causes senescence in EPCs [50], thus we used a short and low-concentration TNFα treatment to minimise chronic effects.

By studying EPC interaction dynamics in healthy BALB/c mouse brains, we observed that some of the fluorescent EPCs integrated into the vasculature within five days. To our knowledge, we are the first to demonstrate that EPC-derived endothelial cells were incorporated into microvessels next to preexisting endothelial cells and displayed continuous TJs between these cells as indicated by claudin-5 immunolabelling. We also found that the number of EPCs observed in brain tissue decreased over time after injection. These EPC-derived, red fluorescent endothelial cells were observed in capillary vessels in the brain parenchyma, usually in small groups of 1–3 cells, but did not form large vascular trees of multiple MAgEC 10.5-derived cells. We found that the literature concerning the effect of EPC in the brain has focused mainly on tissue-level regeneration following ischaemic injury and there are few data available regarding the localisation of injected EPCs in the brain. In contrast to our finding that embryonic EPCs integrated into microvessels in healthy mice, two groups reported that under ischaemic conditions in rats, EPCs differentiated into brain endothelial cells in larger blood vessels. The first report that EPCs integrated into brain vasculature is from 2002 where Zhang et al. injected bone marrow cells from Tie2-LacZ mice in the tail vein of FVB mice that underwent MCAO. They could detect newly differentiated Xgal-positive endothelial cells at the infarct border in 20–50 μm vessels 30 days after ischaemia [51]. Similarly, in Sprague Dawley rats that underwent hypoxia/reperfusion in a 3-vessel occlusion model, CD34 + peripheral blood cells were found to integrate into the walls of 100 μm diameter vessels in the penumbra after 28 days [52]. Data available that show EPCs at microvessels focus on EPC localisation in the ischaemic area but do not examine the integration of EPCs. In CD-1 mice, Fan et al. observed that Dil-labelled EPCs – cultured from human blood – injected through the jugular vein resided in the hypoxic zone at microvessels 24 h after MCAO [34]. In male C57BL/6 mice that underwent permanent MCAO, BrdU-labelled EPCs derived from bone marrow were observed at microvessel walls 3 days after injection in the ischaemic area [53].

Given that the literature data is focused mainly on neuron survival and the vascularisation of postischaemic tissue, we studied EPC integration in the 2VO model using our model EPC line to show that it acts similar to the cell types used by others – in that it alleviates detrimental effects after ischaemic injury in the brain. Our results showed significantly decreased tissue damage based on TTC staining in mice injected with MAgEC 10.5-tomato cells after ischaemic injury. Interestingly, the ipsilateral distribution of MAgEC 10.5-tomato cells observed in healthy brains shifted to a uniform distribution in both hemispheres after 2VO. Moreover, the number of MAgEC 10.5-tomato in brain tissue and more importantly the number of integrated EPCs was significantly increased. The increased occurrence of EPC adhesion and integration into the microvasculature is in good agreement with the observed increase in tissue healing.

By combining senolysis and EPC therapy, we demonstrated that both abt-263 and the combination of dasatinib with quercetin significantly increased the number of MAgEC 10.5-tomato cells that interacted with the vasculature in both young and old mice. The effect was slower to develop in old mice and as the adherent cell counts were already lower, the senotherapy-induced increase appeared low. However, the relative effect of senolysis was similar in old and young mice. Furthermore, we showed that both senolytic treatments increased the number of integrated MAgEC 10.5-tomato in old mice. While the finding that EPCs adhere to old vasculature in lower numbers might seem counterintuitive as a more damaged, old endothelium was expected to recruit more EPCs, similar results have been reported in a skin hypoxia model. The authors explained the observation by decreased hypoxic response of the affected tissue rather than decreased EPC mobilisation or function [54]. The idea that it is not EPC mobilisation that is affected by ageing is supported by the increased number of EPCs in older humans who have higher cerebral small vessel disease scores [55].

## Conclusions

In conclusion, by studying MAgEC 10.5 early embryonic EPC interaction dynamics with mouse brain vasculature, we found that endothelial progenitor cells adhered less to old brain vasculature, which was counteracted by combining EPC injections with senolytic therapy. The interaction of EPCs was increased when the EPCs were activated beforehand, although further optimisation of EPC activation is necessary. A promising candidate for this is TNFα, as it increased both the number of adherent cells and the time the cells remained adherent at the same location in vivo. Furthermore, we demonstrated increased adhesion and integration of EPCs in response to ischaemic injury that may form the basis of the regenerative potential of EPC-based treatment.

Further studies are needed to test whether senolysis-amplified EPC adhesion and integration leads to improved tissue vascularisation and decreased cognitive deficit in ageing. Our results also warrant further studies using these combined therapies in the manifold pathologies where EPCs or senolysis were found effective such as neurodegenerative diseases [56, 57] and age-related pathologies where animal models are available [58]. Sex is another factor to consider in further studies as sex differences can be observed in stroke, endothelial function and EPC numbers [59–61].

## Data Availability

All the data generated or analysed during this study are included in this article. Further details and materials are available upon reasonable request.

## Declarations

### Artificial intelligence (AI)

The authors declare that artificial intelligence is not used in this study.

## Abbreviations

BBB: Blood–brain barrier
EC: Endothelial cell
EPC: Endothelial progenitor cell
MAgEC: Mouse aorta–gonad–mesonephros endothelial cells
TJ: Tight junction
